# Two octave supercontinuum generation in a non-silica graded-index multimode fiber

**DOI:** 10.1038/s41467-022-29776-6

**Published:** 2022-04-19

**Authors:** Zahra Eslami, Lauri Salmela, Adam Filipkowski, Dariusz Pysz, Mariusz Klimczak, Ryszard Buczynski, John M. Dudley, Goëry Genty

**Affiliations:** 1 Photonics Laboratory, Physics Unit, Tampere University, 33014 Tampere, Finland; 2 Łukasiewicz Research Network - Institute of Microelectronics and Photonics, 02-668 Warsaw, Poland; 3 University of Warsaw, Faculty of Physics, 02-093 Warsaw, Poland; 4 Institut FEMTO-ST, Université Bourgogne Franche-Comté CNRS UMR 6174, 25000 Besançon, France

**Keywords:** Solitons, Supercontinuum generation, Fibre optics and optical communications

## Abstract

The generation of a two-octave supercontinuum from the visible to mid-infrared (700–2800 nm) in a non-silica graded-index multimode fiber is reported. The fiber design is based on a nanostructured core comprised of two types of drawn lead-bismuth-gallate glass rods with different refractive indices. This yields an effective parabolic index profile and ten times increased nonlinearity when compared to silica fibers. Using femtosecond pulse pumping at wavelengths in both normal and anomalous dispersion regimes, a detailed study is carried out into the supercontinuum generating mechanisms and instabilities seeded by periodic self-imaging. Significantly, suitable injection conditions in the high power regime are found to result in the output beam profile showing clear signatures of beam self-cleaning from nonlinear mode mixing. Experimental observations are interpreted using spatio-temporal 3+1D numerical simulations of the generalized nonlinear Schrödinger equation, and simulated spectra are in excellent agreement with experiment over the full two-octave spectral bandwidth. Experimental comparison with the generation of supercontinuum in a silica graded-index multimode fiber shows that the enhanced nonlinear refractive index of the lead-bismuth-gallate fiber yields a spectrum with a significantly larger bandwidth. These results demonstrate a new pathway towards the generation of bright, ultrabroadband light sources in the mid-infrared.

## Abstract (web summary)

Two-octave supercontinuum generation in the femtosecond regime from the visible to mid-infrared and self-cleaning dynamics are reported in a non-silica graded-index multimode fiber with nanostructured design and enhanced nonlinearity.

## Introduction

Understanding the physics of complex nonlinear optical systems has been the focus of intense research in recent years and, in this context, the generation of broadband supercontinuum (SC) in graded-index multimode fibers (GRIN MMFs) has attracted particular attention^1–8^. In addition to multimode fibers providing additional degrees of freedom to optimize SC for specific applications, the spatio-temporal propagation in such fibers reveals a rich landscape of nonlinear dynamics, with close links to universal phenomena such as wave turbulence^9–12^.

In contrast to single-mode fibers where the spatial intensity distribution remains constant with propagation, the parabolic index profile of GRIN fibers leads to a periodic self-imaging phenomenon enabling spatio-temporal coupling and mode mixing associated with complex and unique nonlinear dynamics. Spatio-temporal effects that have been observed include among others the generation of multimode solitons^1–3,13,14^, the development of geometric parametric instabilities (GPI)^4–7^, and the formation of GRIN lenses^15,16^. Spatio-temporal dynamics in GRIN silica fibers have been exploited to manipulate the transverse beam profile^4,17^ and, under particular injection conditions, the output beam was observed to exhibit a quasi single-mode profile as the result of nonlinear self-cleaning dynamics^18–21^. Numerical studies have shown that such self-cleaning is a particular feature of pulse propagation in GRIN MMFs^22–24^ associated with strong nonlinear coupling leading to preferential energy transfer to the low-order modes^19,25^. Beam self-cleaning has been reported under various pumping configurations using nanosecond^25^, picosecond^19,26^ and femtosecond^18^ pulses, both in the normal^5,18,27,28^ and anomalous dispersion regime^29^. In addition to being of fundamental importance through its links to universal nonlinear physics, dynamical self-cleaning is also of great practical interest in the development of high-power SC sources.

To date, however, all studies and demonstrations of SC generation in graded-index fibers have been restricted to silica fibers, and bandwidths limited only to the visible and near-infrared spectral regions^18,26,27,29,30^. Yet because of the ability of GRIN MMFs to provide power scaling with a near-Gaussian spatial intensity distribution, there is major interest in extending the GRIN MMF platform into the mid-infrared regime where high spatial beam quality and high power^31^ are required in applications including, e.g., molecular fingerprinting^32^, microscopy^33,34^, medical diagnostics^35,36^, gas monitoring^37,38^, spectroscopy^39,40^, optical coherence tomography^41^ and LIDAR^42^.

Here, we fill this gap and report the generation of a two-octave SC expanding from 700 nm to 2800 nm in a non-silica graded-index multimode fiber. The fiber is designed using two types of lead-bismuth-gallate (PBG) glass rods with different refractive indices drawn to yield a nanostructured core^43–46^. The result is a multimode fiber with an effective parabolic refractive index profile, enhanced nonlinear refractive index, and transmission window up to 2800 nm. Injecting femtosecond pulses into the fiber, we observe the generation of a two-octave SC from 700 to 2800 nm. We conduct a systematic investigation of the SC generating mechanism as a function of pump wavelength, with self-phase modulation (SPM) and GPI seeding the SC generation process in the normal dispersion regime, while in the anomalous regime soliton dynamics and parametric dispersive waves excitation are found to dominate. The relative intensity noise (RIN) has also been characterized in several wavelength bands across the SC spectrum and, under particular controlled injection conditions, we see clear signatures of self-cleaning dynamics with a near single-mode spatial intensity distribution at the fiber output. In order to confirm and interpret our experiments, we perform spatio-temporal 3+1D numerical simulations of the generalized nonlinear Schrödinger equation. Remarkably, the simulations reproduce the spatial intensity distributions measured at the fiber output and confirm the self-cleaning dynamics, with spectra in excellent agreement over the full SC bandwidth. Finally, we perform an experimental comparison with the SC generated in a GRIN silica fiber showing that the spectrum generated in the PBG fiber extends further into the mid-infrared as the result of an enhanced nonlinear refractive index. These results not only open up novel perspectives for the study of nonlinear spatio-temporal instabilities in non-silica graded-index fiber platforms but also provide an avenue for power scaling of SC sources in the mid-infrared.

## Results

### Fiber design characteristics

**Fig. 1:**
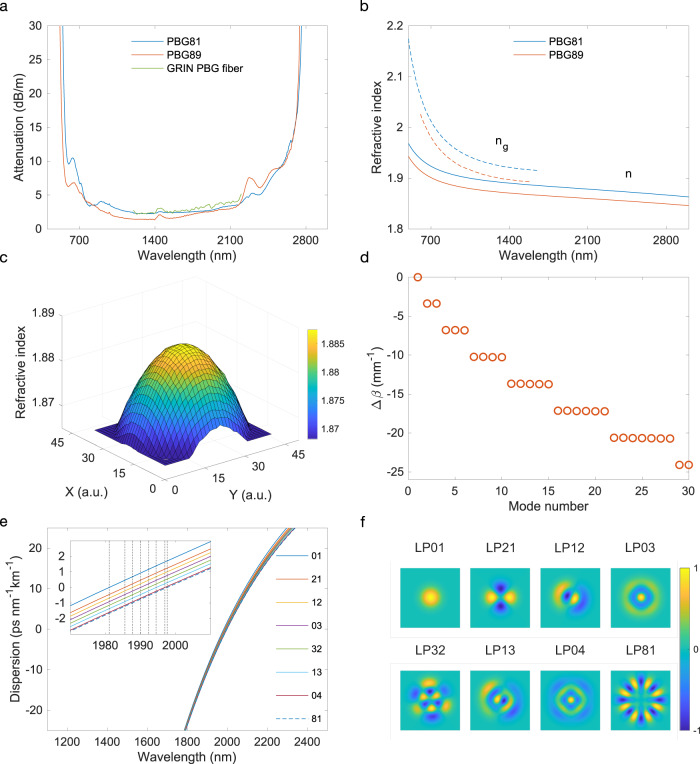
Lead-bismuth-gallate (PBG) graded-index multimode optical fiber characteristics. **a** Experimentally measured attenuation of the bulk PBG-81 (blue) and PBG-89 (red) constituent glasses. The attenuation of the fabricated GRIN fiber is shown in green. **b** Measured group refractive index *n*_*g*_ of PBG-81 (blue dashed) and PBG-89 (red dashed). The calculated refractive index *n* using the extracted Sellmeier coefficients from the measured group index (see Methods) is shown as blue and red solid lines for PBG-81 and PBG-89, respectively. **c** Simulated 3D average refractive index distribution of the fiber structure. **d** Propagation constant difference Δ*β* of the first 30 modes (with respect to the fundamental mode) of the fiber with 80 μm core diameter and refractive-index profile as in **c**. **e** Dispersion profiles of the PBG fiber fundamental and selected higher-order LP modes. The inset in (**e**) shows a magnified view of the dispersion profile near the zero-dispersion region where the vertical dashed lines mark the position of the ZDW. **f** Spatial amplitude distribution corresponding to the dispersion profile of the modes shown in **e**.

The fiber preform was designed to have a parabolic index profile using two types of in-house developed PBG glasses (see Methods for details on the fabrication process). Figure 1 shows the characteristics of the fabricated GRIN PBG fiber with *R* = 40 μm core radius. The relative index difference Δ = (*n*_*co*_−*n*_*cl*_)/*n*_*co*_=0.0101 corresponds to a numerical aperture NA = 0.26 with *n*_*co*_ and *n*_*cl*_ the refractive index at the core center and in the cladding, respectively. Both glasses have a transmission window extending from 400 nm to about 2800 nm limited by OH absorption (Fig. 1a). The measured attenuation of the fabricated fiber (see Methods) is found to be similar to that of the bulk glass, showing that it is primarily the intrinsic attenuation of the glass that limits the fiber transmission. The attenuation is significantly larger than that in silica fiber, yet because the PBG nonlinear refractive index is ten times larger than that of silica^46^ only a short length is needed to observe nonlinear dynamics and massive spectral broadening. The measured refractive index values are around 1.9 (Fig. 1b, see also Methods). The simulated refractive index distribution of the designed fiber is shown in Fig. 1c and exhibits a parabolic variation from the cladding to the core center.

Figure 1d shows the simulated propagation constant Δ*β* of the first 30 modes of the fiber relative to that of the fundamental mode. In contrast to step-index fibers, one can see how the parabolic refractive index profile yields discrete clusters of modes with equally spaced propagation constant and with a cluster population that grows with Δ*β*. This particular feature results in periodic multimodal interference and self-imaging, where the spatial field focuses and defocuses periodically with a period ${z}_{p}=\pi R/\sqrt{2{{\Delta }}}$^7^.

In the experiments reported below, we operate in the regime where the injected peak power is below the critical power for catastrophic self-focusing such that the self-imaging condition is essentially independent of the nonlinear Kerr contribution (see Supplementary Information). Modes within a particular cluster experience identical propagation constant resulting in minimum modal dispersion and walk-off^47^. The propagation constants difference between modes of distinct clusters is large, leading to strong intra-cluster modal interaction but limited inter-cluster mode coupling, such that the fundamental mode is the most stable propagating mode. We also numerically simulated the group velocity dispersion (GVD) of the fundamental and selected higher-order modes vs. wavelengths as shown in Fig. 1e with the corresponding spatial amplitude of the modes illustrated in Fig. 1f. Unlike in step-index multimode fibers^48^, the GVD characteristics of the different modes do not differ significantly with a near-constant zero-dispersion wavelength (ZDW) at ~1980 nm for all modes.

### Experiments and results

**Fig. 2:**
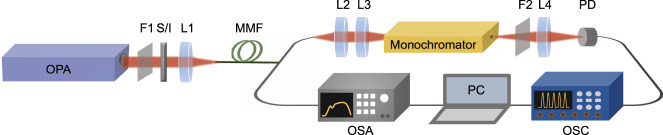
Schematic illustration of the experimental setup for SC generation and intensity noise measurement. *OPA* optical parametric amplifier, *L1–L4* plano-convex lenses, *F1–F2* long-pass filters, *S/I* dichroic filter to select signal/idler, *PD* photodetector, *MMF* multimode fiber, *OSA*, optical spectrum analyzer, *OSC* oscilloscope.

A schematic illustration of our experimental setup is shown in Fig. 2 (see also Methods for additional details). A tunable optical parametric amplifier (OPA) producing 350 fs pulses at a repetition rate of 500 kHz is used as the pump source. The PBG fiber is 20 cm long. Two different optical spectrum analyzers are used to measure the SC spectrum in different wavelength ranges and a monochromator was used to filter out selected wavelength bands and characterize the pulse-to-pulse fluctuations (see Methods for details). The spatial intensity at the fiber output was characterized in the far-field using a beam profiling camera. In all experiments below, we use a very short length of fiber (20 cm) and therefore polarization effects are not expected to play a significant role in the dynamics. Here the injected field is always linearly polarized and a previous study in GRIN silica fibers has shown that, in the nonlinear regime, the degree of linear polarization is generally increased^49^.

**Fig. 3:**
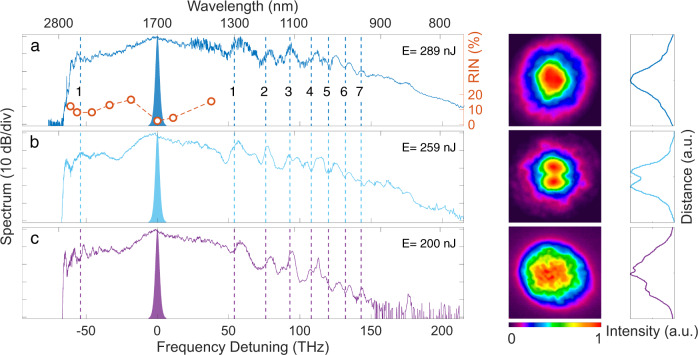
Supercontinuum spectrum and spatial intensity distribution dependence on input beam tilt angle. Left panels: measured supercontinuum spectra at the output of the GRIN PBG fiber with output pulse energy *E* indicated in each subplot. The input beam tilt angle is increased from a to c (see also main text). The vertical dotted lines correspond to the theoretical position of the GPI frequencies with order as shown. The red circles in sub-panel a show the relative intensity noise (RIN) measured for 4000 consecutive pulses in different wavelength bands with 6 nm bandwidth (see Methods). The RIN value of the input pulses at 1700 nm is 1%. Right panels: normalized far-field spatial intensity distribution corresponding to the supercontinuum spectra shown in the left panels. The false-color plots show the 2D intensity distribution while the line plots show the intensity profile along the vertical dimension. a.u: arbitrary units.

We first characterized the output spectrum and spatial intensity distribution at the fiber output for input pulses at 1700 nm in the normal dispersion regime of the fiber. These results are shown in Fig. 3 for three different injection conditions corresponding to increasing tilt between the input beam and the fiber input facet. In all cases, the SC spans from 800 nm to 2800 nm (−40 dB bandwidth) with apparent discrete spectral components on the short-wavelength side. These arise from the nonlinear refractive index grating induced by the Kerr effect and periodic self-imaging along propagation^5,50,51^ that exponentially amplifies small perturbations in frequency bands determined by a specific phasematching condition (see Methods). In contrast to conventional modulation instability in single-mode fibers which is a pure temporal effect restricted to the anomalous dispersion regime, geometric parametric instability (GPI) is a spatio-temporal phenomenon that can occur regardless of the dispersion sign^7^. The bandwidth and amplitude of the GPI sidebands decrease with the order, in agreement with the theory^50^. In principle, GPI leads to the generation of multiple sideband pairs which are widely separated and symmetrically located around the pump. Here, however, because the long-wavelength components fall outside the transmission window of the fiber, we only observe the short-wavelength sidebands except for the first order where we see both spectral components. Note that this was also the case in previous studies in GRIN silica fibers^5,27^.

In order to confirm the origin of the discrete spectral components, we computed the theoretical location of the GPI sidebands for our fiber, and they are shown as dotted lines in Fig. 3 with the sideband order as indicated. For completeness, the experimental GPI high-frequency components and corresponding analytical values up to the seventh order are listed in Table 1 where we see excellent agreement for all generated GPI orders.

**Table 1:** Comparison of theoretically calculated (see Methods) and experimentally measured GPI sideband frequencies.

GPI order							
GPI Sidebands [THz]	1	2	3	4	5	6	7
Theory	53	76	93	108	120	132	143
Experiment (a)	57	79	94	112	125	135	144
Experiment (b)	57	78	92	113	124	135	144
Experiment (c)	58	80	95	114	127	135	144

Similar to conventional modulation instability, an important feature of the geometric parametric instability observed here is that it is seeded by noise outside the pump spectral bandwidth. This is expected to lead to significant shot-to-shot fluctuations yielding a relatively smooth SC spectrum without fine structure when measured with an integrating optical spectrum analyzer^52^. Intensity fluctuations were characterized in selected wavelength bands indicated by the circles in Fig. 3a (see Methods). One can see that the fluctuations are minimal near the pump at 1700 nm and increase significantly away from the pump residue confirming the influence of noise amplification in the SC development.

The spatial intensity profile at the fiber output corresponding to the spectra in Fig. 3 and obtained for different initial excitation conditions shows signatures of self-cleaning dynamics similar to previous observations in silica fibers^18,19,21,25–27^. Specifically, the fiber theoretically supports ~750 transverse modes at 1700 nm, however, when light is injected at normal incidence (Fig. 3a) the measured spatial intensity distribution at the fiber output displays a quasi-Gaussian profile close to the fundamental *LP*_01_ mode. When the light was injected with a small angle with respect to the fiber axis in order to reduce the fraction of energy coupled to the fundamental mode, the intensity profile at the fiber output showed two distinct side-lobes (Fig. 3b) characteristic of the *LP*_11_ mode. Increasing the input angle further to excite a larger fraction of modes at the fiber input (Fig. 3c) yielded an output intensity profile with a fine speckle-like structure indicative of multiple modes contribution. These observations are consistent with nonlinear mode-mixing dynamics mediated by the nonlinear refractive index grating induced along the propagation direction^19,21^. Although the number of modes excited by the injection condition remains essentially constant with propagation^12^, higher-order modes transfer energy via strong nonlinear coupling towards preferential modes as the result of optical wave turbulence^11,12^. This energy flow is similar to wave condensation observed in hydrodynamics and depends on the initial spatial overlap between the input beam and fiber modes^9,11,12,29^. In the case of normal incidence where the input energy is distributed among the lowest-order modes and concentrated around the fiber longitudinal axis, energy preferentially flows from unstable higher-order modes to the fundamental mode^21^, while for a small input tilt the off-axis periodic local intensity oscillation generates an off-axis refractive-index grating overlapping with the *LP*_11_ mode^26^. For a larger tilt, the fraction of energy coupled to higher-order modes at the fiber input is too large for self-cleaning dynamics to fully develop^12^. One can also see that the output SC spectrum changes only slightly with the launching conditions. This can be seen from the spectral bandwidth as well as from the frequencies of the GPI sidebands which remain unchanged in all three cases illustrated. This can be attributed to the fact that the dispersion profile of all modes is nearly identical, leading to dynamics that are essentially independent of the number of excited modes.

**Fig. 4:**
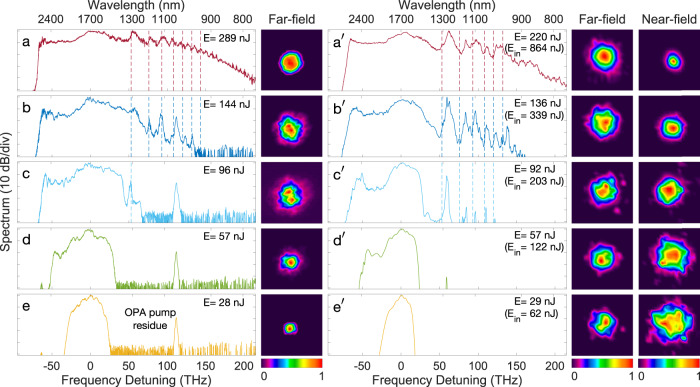
Supercontinuum spectra and corresponding far-field spatial intensity distribution as a function of output energy (E) as indicated and for a pump wavelength of 1700 nm. **a**–**e** Experimental results and ${{\bf {a}}^{\prime}} -{{{{{{{{{\bf{e}}}}}}}}}^{\prime}}$ 3+1D numerical simulations (see Methods for details). The numerical simulations show both the far-field and near-field spatial intensity distributions, and the input energy E_*in*_ is also indicated. The vertical dotted lines mark the theoretical GPI frequencies. The spectral peak at a relative detuning of 111 THz (1040 nm) is a residue from the OPA pump laser.

In order to study the influence of the spatio-temporal dynamics on the SC spectrum and spatial intensity profile at the fiber output, we performed additional measurements, gradually increasing the input peak power for normal incidence launching conditions similar to that in Fig. 3a. The corresponding measured SC spectra and far-field intensity profiles are plotted in Fig. 4. At the lowest injected power value (for which our camera can image the spatial intensity distribution), the nonlinear dynamics are dominated by SPM with near-symmetrical spectral broadening. The corresponding far-field spatial intensity profile is smooth with a narrow diameter, consistent with previous observations in the femtosecond regime^18^. As the injected energy is increased, discrete short-wavelength GPI sidebands develop from noise and the SPM-broadened spectrum extends into the anomalous dispersion regime where soliton dynamics develop. Concomitantly in the far-field, we see an increase in diameter of the beam profile with an apparent contribution from low-order modes. For an input pulse energy of several 100 s of nJ, the beam profile shows a dominant contribution from the *LP*_01_ mode.

To corroborate our experimental observations, we performed numerical simulations using the 3+1D generalized nonlinear Schrödinger equation (see Methods for details) and the results are shown in the right sub-panels of Fig. 4. In principle, one can also use a modal decomposition approach^53^ and the two models are equivalent. However, unlike the 3+1D model which intrinsically includes all propagating modes in the structure, the modal decomposition requires a large number of modes to be included in the simulations for accurate results which can increase significantly the computation time (see Supplementary Information for a comparison between the two approaches). The input energy values in the simulations are adjusted to yield energies at the fiber output similar to those measured in our experiments. We also emphasize that, for the fiber studied here, Raman-induced dynamics are limited because of the reduced Raman contribution arising both from the lower amplitude of the Raman gain in silicate glasses and the short fiber length (see Supplementary Information). The experimentally measured far-field intensity profiles correspond to the Fourier transform of the near field and for completeness, our simulations show both near and far-field distributions. Note that the simulations assume a length of 20 cm which may slightly differ from the experimental fiber length value with a discrepancy that can easily be of the order of one self-imaging period (*z*_*p*_ = 0.9 mm, see Methods). One may therefore expect that the simulated far-field distributions may not exactly match with the experimentally recorded intensity profiles as the near field can vary significantly within one self-imagine period which will also affect the far-field distribution. However remarkably, one can see overall very good agreement between both the experimentally measured and simulated spectra and intensity profiles for all energy values. The simulated evolution of spectra with injected energy follows a similar trend as observed in our experiments, with SPM-dominated broadening. For sufficient increase in injected energy, we observe the emergence of GPI-induced discrete spectral components widely separated from the pump and energy transfer to the anomalous dispersion region with the formation of solitons. At the highest energy values, cross-phase modulation interaction between the SPM spectral component, GPI sidebands, and ejected solitons leads to a quasi-continuous SC spectrum. This scenario is confirmed in the spectrogram animation shown in Supplementary Movie 1 based on a simplified 1+1D model^23^ which reproduces the essential features of the nonlinear propagation (see Supplementary Information). We also see how nonlinear mode-mixing dynamics lead to smoother near-field spatial intensity distribution with decreased beam size.

**Fig. 5:**
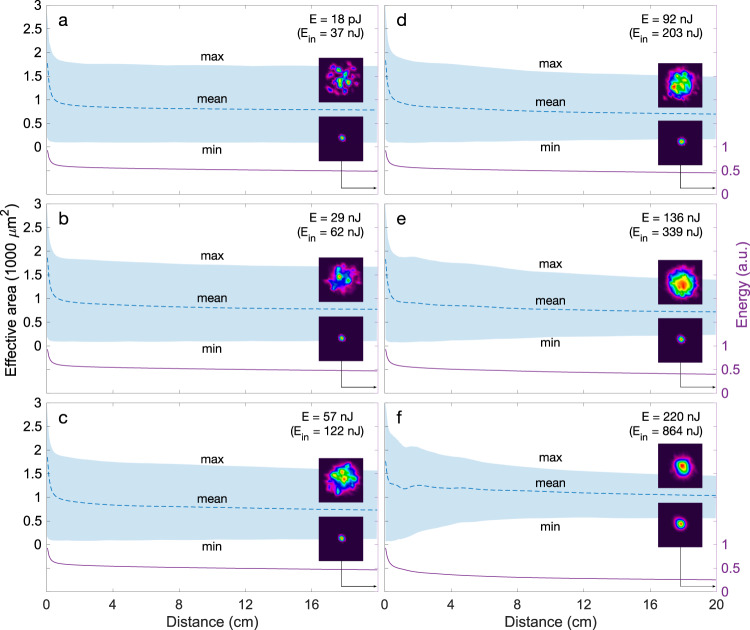
Simulated effective area variations from the linear to nonlinear propagation regime with output (E) and input (E_*in*_) pulse energy as indicated. The shaded region corresponds to the envelope of the effective area fluctuations due to self-imaging with the maximum and minimum values denoted max and min respectively. The blue dashed line corresponds to the mean effective area calculated over one self-imaging period. Inset in a shows the effective area fluctuations inside the envelope over four self-imaging periods. For each injected energy, examples of near-field spatial intensity distributions corresponding to the minimum and maximum beam size are also illustrated at the fiber output. The right axis (purple line) on each sub-panel shows the change in normalized energy as the result of leakage and infrared absorption along propagation with cumulative losses of 3.1, 3.3, 3.3, 3.4, 4.0, and 5.9 dB for **a**–**f**, respectively.

The role of self-cleaning dynamics at higher peak power values is further highlighted in Fig. 5 where we plot the simulated evolution of the beam effective area along with the fiber for the different spectra shown in Fig. 4. For comparison, we also include the evolution in the linear regime in the absence of spectral broadening dynamics. In all cases, the effective area oscillates periodically as the result of the self-imaging dynamics (see inset) and it is this periodic perturbation that leads to the phasematched generation of parametric sidebands^7,50^. We also see that the spatial intensity distribution varies significantly with propagation with clear contributions from higher-order modes in the defocused regions for lower injected energy (Fig. 5a–d). As the injected energy is significantly increased, one can see how the contribution from the higher-order modes decreases dramatically as the result of nonlinear mode mixing with self-cleaning already occurring in the first few centimeters of the fiber (Fig. 5e–f).

**Fig. 6:**
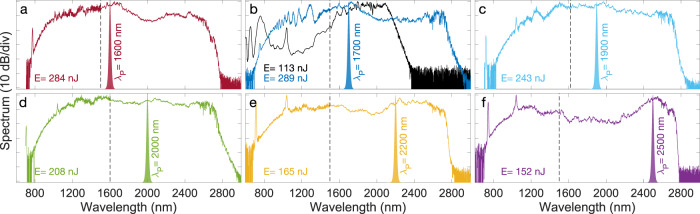
Experimentally measured supercontinuum spectra in 20 cm of the PBG fiber as a function of pump wavelength *λ*_*p*_. **a** *λ*_*p*_ = 1600 nm, **b** *λ*_*p*_ = 1700 nm, **c** *λ*_*p*_ = 1900 nm, **d** *λ*_*p*_ = 2000 nm, **e** *λ*_*p*_ = 2200 nm, and **f** *λ*_*p*_ = 2500 nm. The output pulse energy *E* is indicated in each subplot. The OPA spectrum is also shown for each pump wavelength and the dashed line marks the spectral regions measured using different OSAs (see Methods). The spectral peaks at wavelengths of 750 nm and 1040 nm are residues from the OPA and OPA pump laser, respectively. The black solid line in **b** shows the experimental spectrum obtained in a 20 cm GRIN silica fiber using the same pump wavelength of 1700 nm and injected intensity (see also Methods).

We next investigated the influence of the pump wavelength on the SC spectrum. The results are illustrated in Fig. 6 where we plot the normalized spectra measured at the fiber output corresponding to normal incidence injection conditions that maximized the SC bandwidth. The SC bandwidth is essentially independent of the pump wavelength, with the resulting SC spectrum spanning from 700–800 nm to 2800 nm in all cases, limited on the side of the long-wavelength by the fiber’s intrinsic high attenuation. Interestingly, the GPI sidebands only appear visible in the spectrum when the pump is located in the normal dispersion regime (Fig. 6a–c) but they are not present when the pump is tuned to the anomalous dispersion region beyond 2000 nm (Fig. 6d–f).

This difference can be explained in light of the different SC generating dynamics at play in the two regimes. Specifically, in the anomalous dispersion regime, the initial stage of propagation is dominated by higher-order soliton compression and fission accompanied by the generation on the short-wavelength side of multiple dispersive waves phasematched by the self-imaging dynamics (see Supplementary Information and the spectrogram animation presented in the Supplementary Movie 2). At lower power values the dispersive wave components are clearly visible in the spectrum (see Supplementary Information) but when the injected power is significantly increased, the interaction between the ejected solitons and dispersive waves leads to the broadening of the dispersive wave components and their merging. Finally, we also performed an experimental comparison with the generation of a SC in a commercial GRIN silica fiber (see Methods). The result is illustrated in Fig. 6b as a black solid line for a pump wavelength at 1700 nm. The input energy was scaled to yield an injected intensity identical to the case of the PBG fiber as the two fibers have different core sizes. One can see how the SC spectrum generated in the PBG fiber extends much further to the mid-infrared wavelengths as the result of both a higher nonlinear refractive index and lower dispersion value. We also note that the spectrum extends to shorter wavelengths in the silica fiber with significantly reduced power spectral density below the pump wavelength. This can be explained by the ZDW, which is further detuned from the pump as compared to the ZDW of the PBG fiber.

## Discussion

Despite significant progress in mid-infrared SC generation, there are still important challenges to be overcome in order to obtain SC spectra with characteristics similar to those achieved in the visible/near-infrared. Silica-based platforms exhibit a relatively low nonlinear refractive index which requires significant injected energy to obtain an SC spectrum that extends into the mid-infrared beyond 2 μm. While this has been achieved using very short lengths of single-mode silica fibers^54,55^, it is much less straightforward in multimode fibers due to the large core diameter that reduces even further the nonlinearity. The material aspect is therefore particularly crucial and using non-silica glasses with different refractive indices and a nanostructured core design, we have fabricated a graded-index fiber with an order of magnitude enhanced nonlinearity compared to silica fibers. We have then reported periodic spatio-temporal instabilities and the generation of a broadband SC spanning two-octave up to the mid-infrared. We performed a detailed study of the SC generation process as a function of the pump wavelength and pump power and our results show evidence of nonlinear beam cleaning. Experimental results were compared with spatio-temporal numerical simulations, and the remarkable agreement obtained over the full two-octave bandwidth allows us to confidently interpret the dominant physical mechanisms underlying the SC broadening, even in the complex multimode regime. Some limitations to the achievable spectral bandwidth remain, associated with the strong OH attenuation at longer wavelengths. We believe that improving the purification process of the constituent glasses will allow the extension of the SC spectrum even further, and open up a new avenue for the development of high-brightness SC sources in the mid-infrared.

## Methods

### Fiber fabrication

The GRIN fiber was fabricated using a stack-and-draw method and nanostructured core approach similar to those used to fabricate photonic crystal fibers  and described in ref. ^56^. The gradient index profile was obtained by stacking two types of PBG glass rods (PBG-81 and PBG-89) with distinct refractive indices. The rods have a diameter of 0.35 mm and when stacked together they yielded a structure with a total external diameter of 4.5 cm. The hexagonal structural arrangement of the rods was optimized using a stochastic simulated annealing optimization inspired by the Maxwell-Garnet model^57^. The stacked structure was subsequently drawn in order to fuse the glass rods together to a diameter of about 5 mm. The preform structure was then placed in an external tube acting as the fiber cladding and drawn to a diameter of 125 μm. After the drawing process, the sub-wavelength diameter of the rods effectively yields a gradient index in the core area^16,56^.

### Fiber characterization

The attenuation of the fabricated fiber was characterized over the 1200–2200 nm wavelength range using the cut-back technique, a compact SC source (Leukos SM-30-450), and an optical spectrum analyser (Yokogawa AQ6375B). The initial fiber length of the sample used for the attenuation measurements was 150 cm and cut down to 30 cm in five consecutive cuts. For each fiber length, the spectrum of the transmitted light was measured and averaged over two different cleaves (with minimal change in the fiber length) in order to limit the possible impact of imperfect cleaving. The attenuation shown in Fig. 1 further accounts for Fresnel reflections that were measured from flat-parallel polished glass samples of thicknesses 2 and 4 mm (PBG-81) and 2 and 5 mm (PBG-89). The group index *n*_*g*_ was measured using white-light interferometry and the refractive (phase) index and associated Sellmeier coefficients were then calculated by fitting the first derivative of the Sellmeier equation to the measured group refractive indices^58^

1 $${n}_{g}(\lambda )=n(\lambda )-\lambda \frac{\partial n(\lambda )}{\partial \lambda }=n(\lambda )+\frac{{\lambda }^{2}}{n(\lambda )}\mathop{\sum }\limits_{i=1}^{3}\frac{{B}_{i}{C}_{i}}{{({\lambda }^{2}-{C}_{i})}^{2}}$$

where *n*(*λ*) is the phase refractive index at wavelength *λ* and *B*_*i*_, *C*_*i*_ are the Sellmeier coefficients. The Sellmeier coefficients extracted using this procedure are shown in Table 2 below for both PBG glasses.

**Table 2:** Sellmeier coefficients for PBG-81 and PBG-89 glasses.

	*B*_1_	*B*_2_	*B*_3_	*C*_1_	*C*_2_	*C*_3_
PBG-81	2.01188	0.54673	1.39488	0.01537	0.06355	141.65404
PBG-89	2	0.48632	1.05153	0.0132	0.06741	120

### Experimental setup

The femtosecond laser beam was focused into the fiber using a 5 cm focal length MgF_2_ plano-convex lens resulting in a beam radius of 25 μm (1/e^2^ intensity) at the input facet. The fiber holder was placed on a three-axis precision translation stage to control the input coupling conditions. The fiber was laid straight without any bending or other stress. A dichroic filter was used to select the signal or idler depending on the pump wavelength, and a spectral long-pass filter was inserted to attenuate the OPA pump residue. The maximum throughput was about 45% (calculated as a ratio of output to input power and including coupling efficiency, Fresnel reflection losses, and attenuation along with the fiber). Two optical spectrum analyzers (AQ6315B and AQ6376) were used to measure the SC spectrum in the 350–1700 nm and 1500–3400 nm wavelength range, respectively. Spatial intensity measurements were performed in the far-field at a distance of 2.5 cm using a beam profiling camera (Pyrocam IIIHR).

### GRIN silica fiber

The GRIN silica fiber used for comparison in Fig. 6 is a 20 cm graded-index multimode fiber (Thorlabs M115) with a core diameter of 50 μm, a numerical aperture of 0.2, and zero-dispersion wavelength at ~1300 nm.

### Noise measurements

To characterize the SC pulse-to-pulse intensity fluctuations, light from the fiber output was collimated and directed to a monochromator to filter out wavelength bands with a bandwidth of 6 nm. This bandwidth was selected to yield sufficient energy to be detected as well as to minimize wavelength-averaging. An MgF_2_ plano-convex lens with a 10 cm focal length was used to focus light from the monochromator output to a 15 MHz photodetector (PbSe; PDA10D-EC) connected to a fast 1 GHz real-time oscilloscope (LeCroy WaveRunner 6100 A). Spectral filtering at the monochromator output removed the second-order diffraction of the SC short-wavelength components. The RIN defined as the standard deviation over the mean value was calculated by integrating the voltage of 4000 consecutive pulses after subtracting the noise background.

### Geometric parametric instability sidebands

The frequencies of the geometric parametric instability sidebands *f*_*m*_ of order *m* where *m* = ±1, ±2, ±3... are governed by^7,50^

2 $${(2\pi {f}_{m})}^{2}=\frac{2\pi m}{{z}_{p}{\beta }_{2}}-\frac{2{n}_{2}I{\omega }_{0}}{c{\beta }_{2}}$$

where *n*_2_ is the nonlinear refractive index, *ω*_0_ is the center angular frequency, *I* is the pump pulses intensity, and *β*_2_ is the GVD coefficient at *ω*_0_. The parameter ${z}_{p}=\pi {{{{{{{\rm{R}}}}}}}}/\sqrt{2{{\Delta }}}$ is the self-imaging period with Δ = (*n*_*co*_−*n*_*cl*_)/*n*_*co*_ and *R* the fiber core radius. For our fiber at 1700 nm, *n*_*co*_ = 1.885, *n*_*cl*_ = 1.866, Δ = 0.0101, *R* = 40 μm, *z*_*p*_ = 0.89 mm, *β*_2_ = 6.16 × 10^−26^ s^2^ m^−1^, *n*_2_ = 1.95 × 10^−19^ m^2^ W^−1^.

### Spatio-temporal numerical simulations

We simulate the propagation in the fiber using the 3+1D generalized nonlinear Schrödinger equation which is an extension of the Gross-Pitaevskii equation^13,14^

3 $${\partial }_{z}A+\frac{\alpha }{2}A-i\frac{1}{2{\beta }_{0}}{\nabla }_{T}^{2}A-i\hat{D}A+i\frac{{\beta }_{0}{{\Delta }}}{{R}^{2}}{r}^{2}A=	\, i\gamma (1+{\tau }_{s}{\partial }_{t})\bigg[(1-{f}_{R})| A{| }^{2}A\\ 	+{f}_{R}A\int\nolimits_{-\infty }^{t}{h}_{R}(\tau )| A(t-\tau ){| }^{2}{{{{{{{\rm{d}}}}}}}}\tau \bigg],$$

where the field envelope *A* is expressed in $\sqrt{W}/m$, *r*^2^ = *x*^2^ + *y*^2^, ${\nabla }_{T}^{2}={\partial }_{x}^{2}+{\partial }_{y}^{2}$ is the transverse Laplacian, *α* represents the fiber attenuation, *γ* = *ω*_0_*n*_2_/*c* is the nonlinear coefficient, $\hat{D}={\sum }_{n\ge 2}{(i{\partial }_{t})}^{n}{\beta }_{n}/n!$ is the dispersion operator expanded in terms of Taylor-series coefficients, and *τ*_*s*_ is the shock term. Note that these dimensions and the normalization of *A* used here are different from those conventionally used for the GNLSE with a constant mode area. For completeness, we also investigated the Raman contribution *h*_*R*_ to the overall dynamics. It was modeled as a delayed response using the conventional form

4 $${h}_{R}(\tau )=\left({\tau }_{1}^{-2}+{\tau }_{2}^{-2}\right){\tau }_{1}{e}^{-\frac{\tau }{{\tau }_{2}}}\sin (\tau /{\tau }_{1}),$$

with *τ*_1_ = 5.5 fs, *τ*_2_ = 32 fs, and *f*_*R*_ = 0.05^43^. Its effect on the propagation dynamics was found to be negligible (see Supplementary Information) and therefore was subsequently neglected in the simulations to reduce the computation time. We also use the experimentally measured attenuation (see Fig. 1). Note that the attenuation was measured over the range 1200–2200 nm and we use the bulk glass attenuation for wavelengths outside this range.

The simulations consider pulses of 350 fs duration and a Gaussian temporal intensity profile. Shot noise was added via one-photon-per-mode with a random phase in the frequency domain^52^ and we also include a 0.2% intensity noise in the time domain distributed across the full-time window. The refractive index profile is defined along the radial coordinate by *n*(*r*) = *n*_*co*_−*ar*^2^ for *r* ≤ R and *n*(*r*) = *n*_*cl*_ for *r* > R, where *n*_*co*_ and *n*_*cl*_ are the refractive indices at the center of the core and in the cladding, respectively, and *a* = (*n*_*co*_−*n*_*cl*_)/*R*^2^. Δ is the relative refractive index difference between the core and the cladding and *R* is the core radius.

The Taylor-series expansion coefficients for the dispersion operator are calculated for the refractive index along the fiber longitudinal axis at 1700 nm, and they are *β*_2_ = 6.16 × 10^−26^ s^2^ m^−1^, *β*_3_ = 3.22 × 10^−40^ s^3^ m^−1^, *β*_4_ = −8.96 × 10^−55^ s^4^ m^−1^, *β*_5_ = 2.69 × 10^−69^ s^5^ m^−1^, *β*_6_ = −4.44 × 10^−84^ s^6^ m^−1^, and *β*_7_ = 3.40 × 10^−99^ s^7^ m^−1^. The relative refractive index difference between the core and the cladding Δ = 0.0101, and the nonlinear refractive index *n*_2_ = 1.95 × 10^−19^ m^2^ W^−1^.

At the center wavelength of 2500 nm, the dispersion coefficients are *β*_2_ = −1.30 × 10^−25^ s^2^ m^−1^, *β*_3_ = 8.51 × 10^−40^ s^3^ m^−1^, *β*_4_ = −2.18 × 10^−54^ s^4^ m^−1^, *β*_5_ = 4.55 × 10^−69^ s^5^ m^−1^, *β*_6_ = −5.74 × 10^−84^ s^6^ m^−1^, and *β*_7_ = 3.46 × 10^−99^ s^7^ m^−1^ and the relative refractive index difference between the core and the cladding Δ = 0.0096.

The spatial intensity distribution of the beam is taken to be Gaussian with an input beam radius of 25 μm (1/e^2^ intensity radius). In order to mimic imperfections in the input beam and in the free-space coupling to the fiber, we use a similar approach as in ref. ^19^ and apply to the spatial amplitude distribution a multiplicative phase mask where a random phase shift from (0 to *π*) is added to each spatial grid points. During the very first few centimeters of propagation, large angular frequencies leak to the cladding and a super Gaussian filter is applied to absorb the field at the boundaries of the spatial window. The experimental attenuation of the fiber is similar to that of the bulk constituent glasses over the measured wavelength range (see Fig. 1). We include the attenuation in the simulation model as that of the average of the two glasses since it covers a larger wavelength span. The total losses resulting from leakage to the cladding and infrared absorption are in the range of 3–9 dB depending on the injected power and pump wavelength. The input energy values used in the simulations are adjusted to match the experimentally measured energy at the fiber output.

The simulation grid consists of 16,384 spectral/temporal grid points with a temporal window size of 20 ps and 64 × 64 spatial points with a window size of 160 × 160 μm. A step size of 37 μm was used in the propagation direction. The beam profiles in the far-field are calculated as the Fourier transform from the near-field at the fiber output. The simulated spectra are averaged over 10 realizations with different noise seeds, and the spectra are convolved with a super Gaussian filter with 2 nm bandwidth corresponding to our experimental resolution.

## Supplementary information

Supplementary Information

Simulated spectrogram evolution as a function of distance for a pump wavelength of 1700 nm in the normal dispersion regime of the fiber.

Simulated spectrogram evolution as a function of distance for a pump wavelength of 2500 nm in the anomalous dispersion regime of the fiber.

## Data Availability

Data are available from the corresponding author upon reasonable request.

## Code availability

The simulations code used in this manuscript is available from the corresponding author upon reasonable request.
