# Supplementary material for: Two octave supercontinuum generation in a non-silica graded-index multimode fiber
Source: Nat Commun. 2022 Apr 19;13:2126. doi: 10.1038/s41467-022-29776-6 (PMC9018909; doi:10.1038/s41467-022-29776-6)
Supplement: Supplementary file 1 — Supplementary Information [file 41467_2022_29776_MOESM1_ESM.pdf]

Supplementary Information: Two octave supercontinuum generation in a non-silica graded-index multimode fiber

Zahra Eslami[1], Lauri Salmela[1], Adam Filipkowski[2,3], Dariusz Pysz[2], Mariusz Klimczak[3], Ryszard Buczynski[2,3], John M. Dudley[4], and Goëry Genty[1,*]

[1]Photonics Laboratory, Physics Unit, Tampere University, 33014 Tampere, Finland
[2]Łukasiewicz Research Network – Institute of Microelectronics and Photonics, Al. Lotników 32/46, 02-668 Warsaw, Poland
[3]University of Warsaw, Faculty of Physics, Pasteura 5, 02-093 Warsaw, Poland.
[4]Institut FEMTO-ST, Université Bourgogne Franche-Comté CNRS UMR 6174, 25000 Besançon, France
[*]goery.genty@tuni.fi

Critical power for self-focusing

For sufficiently large peak power, the Kerr nonlinearity can act as a nonlinear lens leading to self-focusing of the beam [1, 2] and catastrophic collapse [3–5]. The critical value of injected power P_{cr} for which Kerr-induced self-focusing occurs can be estimated from [6]

$$P_{\mathrm{cr}} = \alpha \frac{\lambda_0^2}{4\pi n_{co} n_2}, \tag{1}$$

where λ_0 is the central wavelength of the beam, n_{co} the core refractive index of the fiber, n_2 the nonlinear refractive index, and $\alpha \approx 1.9$ for a Gaussian beam. For our fiber, the corresponding critical power value is $P_{\mathrm{cr}} = 1.2$ MW. In our experiments (and numerical simulations), the peak power value is always below this limit due to the significant attenuation resulting from leakage to the cladding in the very first cm of propagation, the rapid temporal broadening when pumping in the normal dispersion regime at 1700 nm and reduced OPA power in the anomalous dispersion regime at 2500 nm. This is also confirmed by the numerical simulation shown in Fig. 5 of the main manuscript.

Effect of Raman response on propagation dynamics

In order to study the effect of the Raman response on the propagation dynamics, we have performed 3+1D GNLSE numerical simulations with and without the Raman contribution included. The Raman response was modeled as a delayed response using the conventional form

$$h_R(T) = \left(\tau_1^{-2} + \tau_2^{-2}\right) \tau_1 e^{-\frac{T}{\tau_2}} \sin(T/\tau_1), \tag{2}$$

with the values τ_1=5.5 fs, τ_2=32 fs corresponding to those measured for PBG glass [7]. The relative contribution of the Raman term to the total nonlinear response is $f_R = 0.05$. The result of the comparison is shown in Supplementary Fig. 1 where we plot the generated supercontinuum (SC) spectra with (right) and without (left) the Raman

term included in the simulations, both for a pump wavelength at 1700 nm in the normal dispersion regime (top row) and 2500 nm in the anomalous dispersion regime (bottom row). One can see that, independently of the pumping regime (normal or anomalous), the Raman response has a negligible effect on the generated SC spectrum. This can be attributed to the relatively low Raman gain of the PBG glasses as compared to silica and the short length of the fiber (20 cm) which limits Raman-induced dynamics.

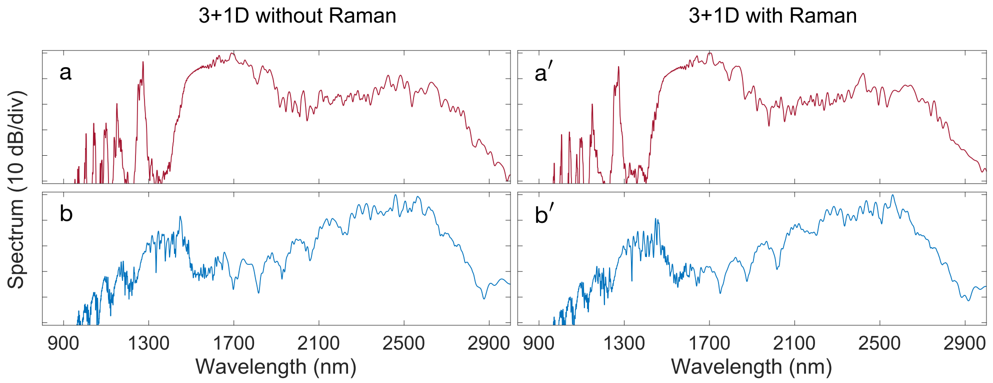

Supplementary Figure 1: Effect of Raman gain on propagation dynamics. Left column: 3+1D simulations without the Raman response included. Right column: 3+1D simulations with the Raman response included. Top row: Pump wavelength at 1700 nm, output energy E=115 nJ (E_{in} =261 nJ). Bottom row: Pump wavelength at 2500 nm, output energy E=35 nJ (E_{in} =168 nJ). The corresponding spectra are generated using identical noise seeds in the spectral and spatial domains.

Modeling comparison

In addition to the 3+1D GNLSE model that was used to produce the results in the main manuscript, we have compared the results with a mode-resolved multimode GNLSE approach (MM-GNLSE) [8] and a simplified (and significantly faster) 1+1D GNLSE model where the self-imaging dynamics are accounted for by a periodic change of the effective area [9].

Unlike the 3+1D GNLSE model that considers the propagation of a spatio-temporal electric field, the MM-GNLSE is based on a modal decomposition of the electric field into spatial modes whose propagation is nonlinearly coupled with the other fiber modes. In this comparison, we only consider the circular LP_{0n} modes, where n is a positive integer. These modes are strongly coupled with the fundamental mode and have a larger overlap with the Gaussian input beam injected to the fiber such that their contribution to the overall supercontinuum development is much more important than that of other modes. In terms of computation time, the MM-GNLSE is dominated by the nonlinear term that involves multiplication of three distinct fields for each mode. The overall computational complexity scales as $\mathcal{O}(P^4)$ for P modes, which results in significant computation time as the number of modes is increased beyond 10 modes [10]. For the numerical simulations shown in the manuscript, the 3+1D model took 12 hours per simulation, which was found to be faster than the mode-resolved simulations when more than 10 modes were included (about 14 hours for 11 modes). However, when only 5 modes are included in the MM-GNLSE model, the computational time dropped to about 45 minutes. Supplementary

Figure 2 shows the comparison between the 3+1D and MM-GNLSE with 5 modes whose initial energy distribution is computed from the overlap integral between the fiber modes and an Gaussian input beam of size as in our experiments. One can see that the SC spectrum simulated from the MM-GNLSE is approaching that of the 3+1D simulations as the number of modes is increased, showing that more than 5 modes should be included for accurate modeling. In the manuscript, we have chosen to use the 3+1D that inherently considers all of the fiber modes, and therefore should model the propagation in a more comprehensive way.

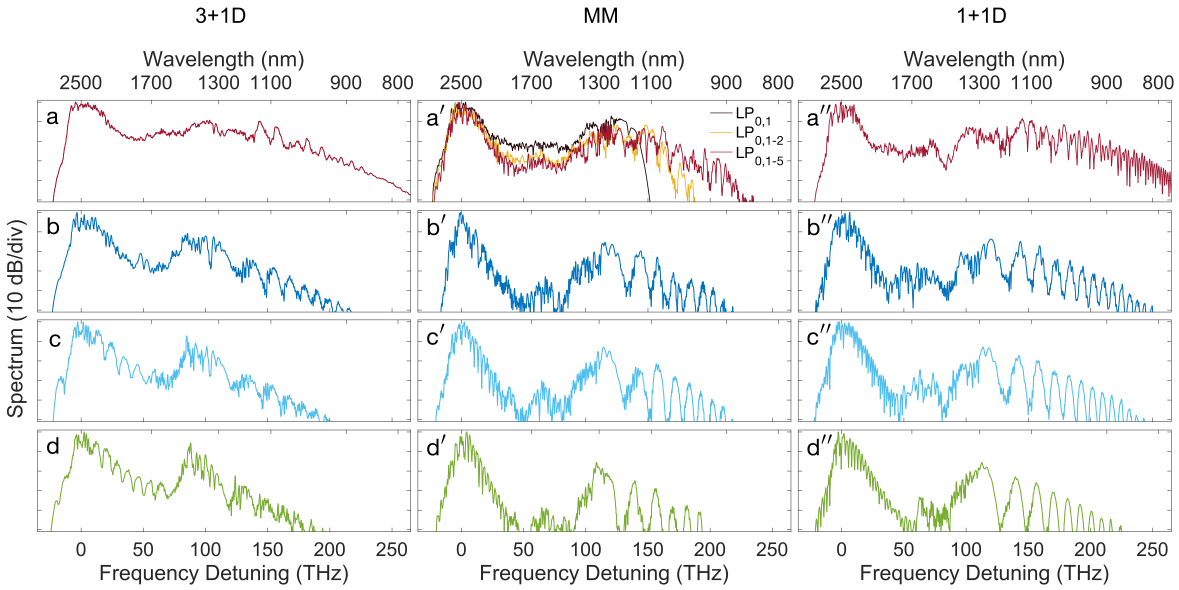

Supplementary Figure 2: 3+1D GNLSE (a-d), MM-GNLSE (a′ to d′) and 1+1D GNLSE (a″ to d'') numerical simulation of supercontinuum spectra as a function of increasing pulse energy a-a″) E_{in} = 140 nJ, b-b″) E_{in} = 70 nJ, c-c″) E_{in} = 59 nJ, d-d″) E_{in} = 48 nJ (for a fixed length of 20 cm and pump wavelength of 2500 nm). The corresponding output energies for the 3+1D GNLSE are 57, 34, 30 and 25 nJ, for the MM-GNLSE with 5 modes 70 (68 and 64 for two and one modes), 41, 36 and 30 nJ, and for the 1+1D GNLSE 59, 38, 34 and 29 nJ, respectively. The injected beam was Gaussian (both in the spatial and temporal domains) without phase noise for the 3+1D simulations. The MM-GNLSE simulations include the 5 lowest LP_{0n} modes. In a' we also show the SC spectrum obtained considering only the fundamental mode LP_{01} (black line) and the two lowest LP_{0n} ($n = 1, 2$) modes (yellow line). The spectra are averaged over 10, 10 and 50 realizations for the 3+1D GNLSE, MM-GNLSE and 1+1D GNLSE simulations, respectively. Shot noise was added in MM-GNLSE and 1+1D GNLSE models via a one-photon-per-mode with random phase in the frequency domain [11].

In the simpler 1+1D approach, the beam spot size (1/e intensity radius) along the fiber can be written as [9]

$$a^2(z) = a_0^2[\cos^2(\sqrt{g}z) + C\sin^2(\sqrt{g}z)], \tag{3}$$

where a_0 is the input beam radius (1/e intensity waist), $g = 2\Delta/R^2$, $\Delta = (n_{co}^2 - n_{cl}^2)/2n_{co}^2$ is the relative refractive index difference, and $C = (1-p)/(\beta_0 a_0^4 g) \approx 1/\beta_0 a_0^4 g$ under stationary self-imaging conditions. The nonlinear coefficient γ of the fiber can be calculated

through the z-dependent beam size as

$$\gamma(z) = \frac{\omega_0 n_2}{c A_{\text{eff}}(z)} = \frac{\omega_0 n_2}{2\pi c a^2(z)}, \tag{4}$$

where $A_{\text{eff}}(z)$ is the effective area of the beam which can be approximated by $2\pi a^2(z)$. We consider an input beam size of 25 μm (1/e^2 intensity radius), fiber core radius of $R = 40$ μm, and nonlinear refractive index n_2 of 1.95×10^{-19} m^{2}W^{-1}. The simulations use up to 65536 spectral/temporal grid points with temporal window size of 80 ps, spectral resolution of 12.5 GHz and a step size of 6.7 μm (30,000 steps). Shot noise is added via one-photon-per-mode with random phase in the frequency domain. In the comparison, no input phase noise is added to the spatial amplitude distribution for the 3+1D model. Supplementary Figure 2 compares the average SC spectrum for increasing input peak power and a pump wavelength of 2500 nm. The SC spectra were convolved with a super Gaussian filter with 2 nm bandwidth and averaged over 10, 10 and 50 realizations, respectively. In agreement with previous studies [9,12], we see relatively good correspondence between the models. The 1+1D model reproduces the main features and can be conveniently used to verify the propagation dynamics that leads to the supercontinuum development while saving considerable computing time and memory, and this is the model we use in the Supplementary Movies showing the time-frequency spectrogram evolution along propagation. Note that including spatial input phase noise in the 3+1D model (as was done for the simulation results shown in the main manuscript) leads to a fraction of the energy to leak to cladding and a reduced supercontinuum spectral bandwidth as compared to when spatial noise is not added (and also the 1+1D model). This can be compensated for when spatial noise is added by adjusting the injected peak power to yield an output energy identical to the noiseless case. We also emphasize that the energy loss observed between the input and output is essentially dominated by the fiber attenuation in all three models.

Power dependence of the supercontinuum spectrum for 2500 nm pump wavelength

We characterized the supercontinuum spectrum generated as a function of injected peak power when the OPA was tuned to 2500 nm in the anomalous dispersion regime of the fiber. The results are shown in Supplementary Fig. 3. In this case the generating mechanism is triggered by higher-order soliton compression and fission with multiple dispersive waves emission that are manifested as discrete spectral components in the normal dispersion region. This scenario is confirmed by the simulated spectrogram evolution along propagation (using the simplified 1+1D model) and shown in the Supplementary Movie 2. Note that unlike the geometric parametric instabilities observed in the normal dispersion regime and that are seeded by noise, the multiple dispersive waves emission mechanism is a coherent process seeded by the spectral components of the compressed higher-order soliton spectrum [13, 14]. The theoretical location of the dispersive wave angular frequencies ω_m where m is an integer ($m = 0, \pm 1, \pm 2, \pm 3...$) can be determined from the following phasematching condition [15, 16]:

$$\sum_{k \geq 2} \frac{\beta_k}{k!} (\omega_m - \omega_0)^2 - \frac{\gamma P_p}{2} = \frac{2\pi m}{z_p} \tag{5}$$

where β_k represent the Taylor series expansion dispersion coefficients at the pump angular frequency ω_0, γ is the nonlinear coefficient and P_p the peak power at the point of maximum higher-order soliton compression. $z_p = \pi R/\sqrt{2\Delta}$ is the self-imaging period with $\Delta = (n_{co} - n_{cl})/n_{co}$ and R the fiber core radius. At 2500 nm $n_{co} = 1.872$, $n_{cl} = 1.854$, $\Delta = 0.0096$, R=40 µm, $z_p = 0.91$ mm. The values of γ and P_p at the point of maximum soliton compression are given in the caption of Supplementary Fig. 3. At low power values, the dispersive waves are clearly apparent in the spectrum and their frequencies are in good agreement with the theoretical predictions indicated by the vertical dashed lines. The residual discrepancy arises from cross-phase modulation with the solitonic components inducing a shift in the dispersive waves position. In principle and in contrast to dispersive waves emitted in single-mode fibers, the periodic perturbation arising from self-imaging can produce dispersive waves components both on the short and long wavelengths side of the spectrum. However, here the long wavelengths components are not observed due to the strong glass absorption beyond 2800 nm. As the peak power is increased, the number of dispersive waves components increases. For the largest injected peak power value interactions via cross-phase modulation between the large number of solitons ejected from the fission process and the dispersive waves leads to the merging of the latter yielding a quasi-continuous supercontinuum spectrum.

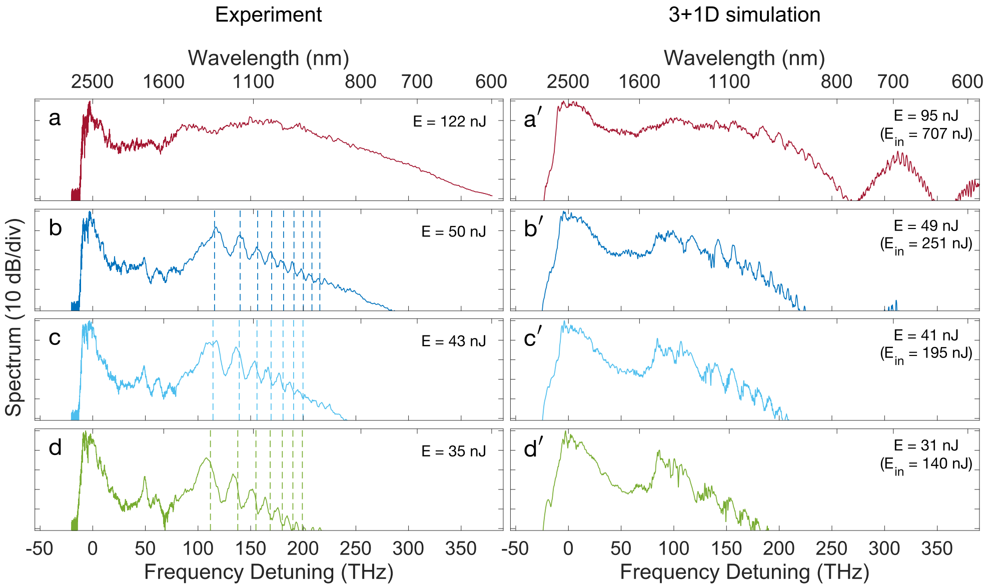

Supplementary Figure 3: Experimental (a-d) and numerical (a' to d') supercontinuum spectra generated in 20 cm of the fiber for a pump wavelength at 2500 nm and increasing input energy. The input pulse energy E_{in} in the simulations was adjusted to yield an output energy E close to that measured in the experiments. The simulated spectra are averaged over 10 realizations with different spectral and spatial noise seeds. The vertical dashed lines indicate the theoretical frequency of the radiated dispersive waves. Note that in calculating the wavelength of the dispersive waves we have used the nonlinear coefficient and peak power values at the point of maximum compression taken from the numerical simulations: From b to d, $\gamma = 2.5 \times 10^{-3}$ m^{-1}W^{-1} and $P_p = 1.3$ MW, $\gamma = 2.4 \times 10^{-3}$ m^{-1}W^{-1} and $P_p = 1.1$ MW, $\gamma = 2.4 \times 10^{-3}$ m^{-1}W^{-1} and $P_p = 700$ kW.

Spectrogram animations

The Supplementary Movies show the numerically simulated spectrogram evolution as a function of propagation distance for a pump wavelength at 1700 nm and 2500 nm, based on the 1+1D model. The spectrogram is calculated as

$$S(\omega, \tau, z) = \left| \int_{-\infty}^{+\infty} g\,(T - \tau)\, E(z, T) \exp(-i\omega T) dT \right|^2 \tag{6}$$

where $E(z, T)$ represents the time-dependent complex electric field at propagation distance z and $g(T - \tau)$ is a gate function with delay value τ. The spectrogram animation shows the spectra of a series of time-gated portions of the field as a function of propagation coordinate. In our calculation of the spectrogram, we used a hyperbolic-secant gate function of 100 fs duration (full-width at half-maximum).

References

[1] Chiao, R. Y., Garmire, E. & Townes, C. H. Self-trapping of optical beams. *Physical Review Letters* **13**, 479 (1964).

[2] Kelley, P. Self-focusing of optical beams. *Physical Review Letters* **15**, 1005 (1965).

[3] Gaeta, A. L. Catastrophic collapse of ultrashort pulses. *Physical Review Letters* **84**, 3582 (2000).

[4] Vaziri, M. R. Describing the propagation of intense laser pulses in nonlinear Kerr media using the ducting model. *Laser Physics* **23**, 105401 (2013).

[5] Moll, K., Gaeta, A. L. & Fibich, G. Self-similar optical wave collapse: observation of the townes profile. *Physical Review Letters* **90**, 203902 (2003).

[6] Fibich, G. & Gaeta, A. L. Critical power for self-focusing in bulk media and in hollow waveguides. *Optics Letters* **25**, 335–337 (2000).

[7] Sobon, G. *et al.* Infrared supercontinuum generation in soft-glass photonic crystal fibers pumped at 1560 nm. *Optical Materials Express* **4**, 7–15 (2014).

[8] Poletti, F. & Horak, P. Description of ultrashort pulse propagation in multimode optical fibers. *JOSA B* **25**, 1645–1654 (2008).

[9] Conforti, M., Arabi, C. M., Mussot, A. & Kudlinski, A. Fast and accurate modeling of nonlinear pulse propagation in graded-index multimode fibers. *Optics Letters* **42**, 4004–4007 (2017).

[10] Wright, L. G. *et al.* Multimode nonlinear fiber optics: massively parallel numerical solver, tutorial, and outlook. *IEEE Journal of Selected Topics in Quantum Electronics* **24**, 5100516 (2017).

[11] Dudley, J. M., Genty, G. & Coen, S. Supercontinuum generation in photonic crystal fiber. *Reviews of Modern Physics* **78**, 1135 (2006).

[12] Wright, L. G., Wabnitz, S., Christodoulides, D. N. & Wise, F. W. Ultrabroadband dispersive radiation by spatiotemporal oscillation of multimode waves. *Physical Review Letters* **115**, 223902 (2015).

[13] Akhmediev, N. & Karlsson, M. Cherenkov radiation emitted by solitons in optical fibers. *Physical Review A* **51**, 2602 (1995).

[14] Erkintalo, M., Xu, Y., Murdoch, S., Dudley, J. M. & Genty, G. Cascaded phase matching and nonlinear symmetry breaking in fiber frequency combs. *Physical Review Letters* **109**, 223904 (2012).

[15] Conforti, M., Trillo, S., Mussot, A. & Kudlinski, A. Parametric excitation of multiple resonant radiations from localized wavepackets. *Scientific Reports* **5**, 9433 (2015).

[16] Eftekhar, M., Lopez-Aviles, H., Wise, F., Amezcua-Correa, R. & Christodoulides, D. General theory and observation of cherenkov radiation induced by multimode solitons. *Communications Physics* **4**, 137 (2021).
